# Gauging the Effectiveness of Training Sessions Among Nurses Regarding Biomedical Waste Management: A Quasi-Experimental Study From a Developing Country

**DOI:** 10.7759/cureus.12196

**Published:** 2020-12-21

**Authors:** Tahira Shaheen, Mansoor Ghani, Samina Kausar

**Affiliations:** 1 Nursing, University of Health Sciences, Lahore, PAK; 2 Internal Medicine, University of Health Sciences, Lahore, PAK

**Keywords:** health-care workers, biomedical waste management

## Abstract

Introduction

Biomedical waste management (BWM) plays a crucial role in maintaining human and environmental health. Unfortunately, health-care workers (HCWs) lack the essential awareness concerning BWM and there is a dire need to adopt different strategies to improve their practices. This research aims to evaluate the effectiveness of training sessions among HCWs regarding biomedical waste management using a quasi-experimental study design.

Methods

This quasi-experimental study included a total of 64 nurses, selected with a systematic random sampling technique. Three days of structured training sessions were organized in the morning and evening shifts. Pertinently, pre-test and post-test were organized before and after the end of training sessions. Practices of nurses regarding BWM were also assessed before the training and after one month of training with the aid of a checklist.

Results

The low pre-test scores of the study participants elucidated insufficient knowledge regarding various aspects of BWM. After the three days of the structured training sessions, the analysis of post-test scores elucidated a marked improvement in the knowledge of the study participants. The practices of nurses regarding BWM were inappropriate; however, one month after the training sessions, the re-evaluation of practices showed a significant improvement.

Conclusion

The study showed that nurses had poor knowledge regarding BWM and were significantly improved on teaching interventions. An essential knowledge regarding BWM is therefore very useful for HCWs to protect themselves from infectious diseases. The inclusion of regular training sessions in the teaching curriculum can ensure adherence to guidelines for appropriate BWM. Assurance of ideal practices for BWM plays a key role in the prevention of nosocomial infection among HCWs.

## Introduction

Biomedical waste management (BWM) is an emerging global public health concern that requires immediate interventions. There are improved practices among health-care workers (HCWs) regarding BWM in developed countries but developing countries considerably lag [1]. BWM practices in Pakistan are non-compliant to the standards of the World Health Organization [1,2]. This results in an exponential increase in the incidence of hepatitis B, C, and other infectious diseases among patients and HCWs [2]. An observational study represented that nurses were deficient in knowledge of biomedical waste management therefore practices are not appropriate [3]. Another study elucidated that biomedical waste harms the community, environment, and essential HCWs (nurses and paramedical staff) [4]. Furthermore, studies have advocated that color-coding awareness and BWM among HCWs remains inadequate [3,5]. Henceforth, appropriate training workshops become an essential requirement for ideal BWM. Biomedical waste produced in the course of health care activities carries a higher potential for infection to HCWs than other waste and training regarding biomedical waste management should be emphasized [6]. Lack of proper and complete knowledge about BWM leads to flawed practices of appropriate waste disposal.

Biomedical waste is regarded as the second dangerous waste in the world and requires proper disposal with safety measures according to international guidelines [7]. It has been estimated that each hospital bed generates nearly 1.25 kg of biomedical waste every day [8]. These statistics further necessitate the emphasis on appropriate BWM so that the risk of acquiring nosocomial infections can be minimized. A study advocates that essential HCWs in developing countries especially nurses have poor awareness about the risks of biomedical waste and resultantly succumb to various infectious diseases [9]. Furthermore, the same study averred that orientation and reorientation of training sessions should be organized for HCWs especially nurses with strict implementation of BWM guidelines so that they are protected from a myriad of infectious diseases [9].

The significance of training about BWM with training sessions needs to be accentuated as a deficiency of proper and complete knowledge about BWM influences practices of proper waste disposal [10]. This will equip the HCWs to prevent themselves from the hazards of biomedical waste. Studies have found a strong association between good knowledge and adherence to standard practices for BWM among nurses [9,11]. Moreover, nurses with higher education have a better awareness of national and international enhancement in BWM [12]. The literature also shows that periodic repeated training plays an important role in the improvement of knowledge and awareness level of hospital staff about BWM [2,13].

This study aims to evaluate the effectiveness of training sessions among nurses regarding BWM using a quasi-experimental study design. We also aim to assess the knowledge of nurses before and after the training sessions. Additionally, we also target to observe the improvement in practices of nurses regarding BWM before and after one month of the training sessions.

## Materials and methods

This quasi-experimental was conducted at the Institute of Nursing, University of Health Sciences (UHS), Lahore, Pakistan. with the collaboration of Lady Aitchison Hospital, Lahore, Pakistan. A total of 64 nurses working in Lady Aitchison Hospital Lahore were selected via systemic random sampling technique. Nurses with below one year of working experience were excluded from the study. Furthermore, nursing superintendents and nursing instructors working in Lady Atchison Hospital Lahore were also subjected to exclusion.

The instrument used in this study had three components. The first portion enquired regarding the demographic profile of the participants. The second component had questions regarding the knowledge of BWM. While the third component dealt with the observation checklist which assessed the practices of the study participants. Both knowledge and practices were assessed before and after one month of training sessions. These training sessions continued for three days with four hours of duration per day. Training session for three days of four-hour duration per day.

Test of knowledge was assessed by self-structured questionnaire and practices of participants were assessed by checklist before the start of training and then after the training. The maximum score was 25 and the minimum was zero. For each correct response, one mark was given and zero marks for each wrong answer. Scores between zero to 10 showed a poor level of knowledge, scores of 11-18 elucidated a good level of knowledge while scores above 19 showed an excellent level of knowledge regarding BWM. The same questionnaire was used to assess the sustainability of knowledge among study participants after the end of the training sessions. Practices regarding BWM were also assessed with a checklist before and after one month of training.

The training session was carried out in two groups. Each group consisted of 32 nurses that were trained in the morning and afternoon sessions. Each session was interactive and had practical demonstrations on BWM. All participants were involved during the discussion. The learning objectives of the training sessions were elaborated to be the participants and by the end of the training, all nurses must be able to: (1) define and classify biomedical waste; (2) list the sources of biomedical waste; (3) recognize the need for BWM; (4) use of the process of segregation, collection, storage, transportation, treatment, and final disposal; (5) recognize the problems associated with biomedical waste; (6) practice the BWM rules in Pakistan; (7) recognize the categories of biomedical waste and handling of waste according to the BWM rules; (8) identify the color coding and type of container for the disposal of waste; and (9) explain the role of the nurse in BWM. 

Questionnaires were given to the participants as a pretest, which was collected after 30 minutes. Pertinently, three days training session was given to the participants which include lectures, discussions, demonstrations, and return demonstrations. After three days of sessions, the participants were assessed using a post-test survey. Feedback was also received from the study participants.

The data analysis was carried on the Statistical Package for Social Sciences (SPSS) software version 23 (IBM Corp., Armonk, NY). The data were presented in the form of percentages and frequencies for categorical variables. Mean with standard deviation were calculated for quantitative variables. Paired t-test and chi-square test were used for comparison of scores of knowledge and practices. A p-value of less than 0.05 was considered statistically significant.

## Results

In the current study analyzing 64 nurses, a total of 87.50% of nurse’s education had completed matriculation while 12.50% had a bachelor's degree. In the study sample of 64 nurses, 10 (15.63%) had a working experience of one to three years, 15 (23.44%) had three to five years of working experience, 23 (35.94%) had an experience of five to seven years, while 16 (25%) nurses had a working experience of more than eight years. The biomedical waste handler nurses were assembled into three categories according to their knowledge scores. There was a significant improvement in the knowledge scores of the nurses after the sessions. This is elucidated in Table 1.

**Table 1:** A tabulation of pre- and post-training assessment scores.

Knowledge score	Before training		After training	
	Frequency	Percentage	Frequency	Percentage
Poor (0-10)	04	6.25%	00	0%
Good (11-18)	45	70.32%	02	3.13%
Excellent (19-25)	15	23.43%	62	96.87%
Total	64	100%	64	100%

Comparison of mean knowledge scores before and after the end of training sessions showed a significant difference between pre- and post-test scores. This is shown in Table 2.

**Table 2:** Comparison of pre- and post-test scores of the participants. *Paired t-test.

Parameter	Mean	Standard deviation	P-value*
Pre-test score	15.75	4.254	0.001
Post-test results	22.36	1.971	

**Figure 1:**
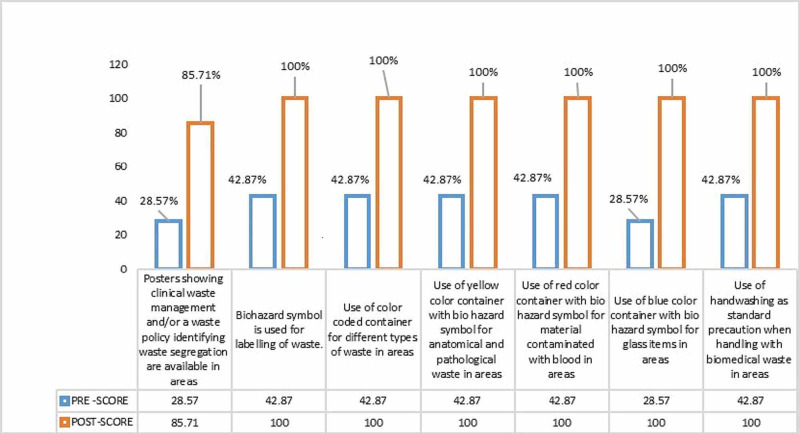
An elucidation of the practice of nurses before and after one month of training sessions.

Evaluation of practices showed that the majority of the nurses exhibited wrong practices before training sessions while after one month of these training sessions the practices were found to be according to the BWM guidelines. Before training, most of the nurses didn’t know about the biohazard symbol and color-coding used for biomedical waste management but after one month of these three days training sessions, the practices were improved. A graphical representation of the evaluation of practices regarding BWM before and after training are shown in Figure 1.

## Discussion

There was a significant improvement of knowledge after the training session with no participants in the poor knowledge level category. Another study also revealed that the biomedical waste handlers showed significant improvement regarding BWM after training [14]. A meta-analysis elucidated that nurses have inadequate knowledge regarding the BWM and necessitated the need for more cross-sectional studies that assess the knowledge and practices of HCW so that overall practices can be improved [14]. Our scale-covered all aspects of the BWM and showed that most nurses improved their knowledge from poor to excellent category. This demonstrates that teaching sessions were effective in improving the overall knowledge of the study cohort.

The first section of the questionnaire included two questions about the definition of biomedical waste and the name of diseases caused by infectious waste. This result showed a statistically significant difference in pre- and post-test and 95% of nurses gave correct answers after the session. Similarly, more studies also showed significant improvement in BWM among nurses after the conduction of appropriate training sessions [13,15]. Pertinently, 50% of the nurses had adequate knowledge regarding the collection and segregation of waste before the start of the training sessions which improved to 97%. A study revealed that all nurses had poor knowledge regarding the diseases which can be transmitted via biomedical wastes and it significantly improved to an excellent level of knowledge after the educational intervention [10]. These results are consistent with our study where approximately 70% of nurses knew diseases spread by infectious waste which was enhanced to 95% after the sessions [10].

The awareness of color coding is very important for the nurses because segregation at the point of generation in specified color containers was done by biomedical waste handlers [12,16]. All nurses should know about the waste and specific color where it can be collected from the wards and operation theaters [2]. In this study, only 40% of the nurses had adequate knowledge of color-coding which was also improved after training. In another study, the same results showed a significant association between waste segregation and training of BWM among HCWs [16]. Therefore, training is an effective method that can make an appreciable increase in the knowledge about the color coding of biomedical waste.

Sterilization is very important during the handling of instruments after the use. Nurses showed almost 60% results in the pretest and 78% in the post-test, which is not a remarkable improvement in knowledge. Existing knowledge is also satisfactory but improvement needs are compulsory on the topic of sterilization. Another study showed similar results and emphasized that knowledge about standard precautions is already adequate among nurses which can be improved to an excellent level with the use of different educational training [3]. Many other studies in the literature are consistent with our findings and agreed that raining sessions significantly improve the knowledge and practices of HCWs regarding BWM [3,17].

Most of the nurses in our study showed inappropriate practices regarding BWM before the training session. All the areas were improved after the demonstration and return demonstration which was assessed after one month of this training session. Six out of seven components showed 100% correct practices after a one-month assessment. Another study observed in their study that no one scored excellent before the intervention of the training program [18]. It showed that 39 (20.8%) of health providers had good practices and the majority 148 (79.1%) had poor practices before the educational intervention [18]. Post educational intervention had shown enhancement in practices, which displayed that the educational intervention was effective in refining knowledge [17,18].

The present study showed that there was an improvement in knowledge and practices both than pre-assessed knowledge and practices. Some areas showed insignificant results and need proper attention to those points to increase their knowledge and the compliance of practices as well as standard precautions and personal protective measures to reduce the diseases and infection rate.

## Conclusions

The knowledge of nurses regarding BWM was extremely insufficient which significantly improved with the conduction of teaching sessions. The training sessions regarding BWM are exceedingly necessary and their importance cannot be overemphasized. Knowledge regarding BWM is very essential to prevent health care providers to protect themselves from infectious diseases. Training sessions improved the overall knowledge and practices of nurses regarding BWM. Safe practices with adherence to international guidelines for BWM plays a crucial role in limiting the transmission of fatal infectious diseases among HCWs.
